# Increased Oxidative Stress Markers in Cerebrospinal Fluid from Healthy Subjects with Parkinson’s Disease-Associated *LRRK2* Gene Mutations

**DOI:** 10.3389/fnagi.2017.00089

**Published:** 2017-04-03

**Authors:** David A. Loeffler, Andrea C. Klaver, Mary P. Coffey, Jan O. Aasly, Peter A. LeWitt

**Affiliations:** ^1^Department of Neurology, Beaumont Hospital-Royal Oak, Beaumont Health, Royal OakMI, USA; ^2^Department of Biostatistics, Beaumont Hospital-Royal Oak, Beaumont Health, Royal OakMI, USA; ^3^Department of Neurology, St. Olav’s HospitalTrondheim, Norway; ^4^Department of Neurology, Henry Ford Hospital, DetroitMI, USA; ^5^Department of Neurology, Wayne State University School of Medicine, DetroitMI, USA

**Keywords:** 8-isoprostane, 8-hydroxydeoxyguanosine, LRRK2, oxidative stress, Parkinson’s disease, total antioxidant capacity

## Abstract

Mutations in the leucine-rich repeat kinase 2 (*LRRK2*) gene are the most frequent cause of inherited Parkinson’s disease (PD). The most common PD-associated *LRRK2* mutation, G2019S, induces increased production of reactive oxygen species *in vitro*. We therefore hypothesized that individuals with PD-associated *LRRK2* mutations might have increased concentrations of oxidative stress markers and/or decreased total antioxidant capacity (TAC) in their cerebrospinal fluid (CSF). We measured two oxidative stress markers, namely 8-hydroxy-2′-deoxyguanosine (8-OHdG) and 8-isoprostane (8-ISO), and TAC in CSF from *LRRK2* mutation-bearing PD patients (*LRRK2* PD = 19), sporadic PD patients (sPD = 31), and healthy control subjects with or without these mutations (*LRRK2* CTL = 30, CTL = 27). 8-OHdG and 8-ISO levels were increased in *LRRK2* CTL subjects, while TAC was similar between groups. 8-ISO was negatively correlated, and TAC was positively correlated, with Montreal Cognitive Assessment scores in *LRRK2* PD, *LRRK2* CTL, and CTL subjects. Correlations in both groups of PD patients between the two oxidative stress markers and Unified Parkinson Disease Rating Scale Total scores were weak, while TAC was negatively correlated with these scores. These findings suggest that oxidative stress may be increased in the CNS in healthy individuals with PD-associated *LRRK2* mutations. Further, TAC may decrease in the CNS with the progression of PD, and when cognitive impairment is present regardless of the presence or absence of PD.

## Introduction

Parkinson’s disease (PD) is most commonly a sporadic disorder, but up to 10% of PD cases are associated with genetic mutations (Van Den Eeden et al., 2003). The main neuropathological finding in PD is extensive loss of dopaminergic neurons whose cell bodies are located in the substantia nigra pars compacta (SNC) and whose axons project to the striatum (Davie, 2008). Increases in lipid, protein, and nucleic acid oxidation have been reported in the PD SNC (Dexter et al., 1994; Yoritaka et al., 1996; Alam et al., 1997; Floor and Wetzel, 1998; Zhang et al., 1999) suggesting that oxidative stress (overproduction of reactive oxygen and nitrogen species relative to their detoxification mechanisms [Wang and Michaelis, 2010]) may play a role in the loss of dopaminergic neurons (Götz et al., 1990; Spencer et al., 1994; Dias et al., 2013). Factors thought to contribute to increased oxidative stress in the PD brain include mitochondrial dysfunction (Schapira, 2008), dysregulated iron metabolism (Dexter et al., 1989), neuroinflammation (Peterson and Flood, 2012), decreased antioxidant levels (Perry et al., 1982), and elevated production of H_2_O_2_ and reactive oxygen species (ROS) as a consequence of increased dopamine turnover (Fahn and Cohen, 1992).

Mutations in the gene encoding for leucine-rich repeat kinase 2 (*LRRK2*) are the most frequent known cause of inherited PD in most populations (Vilas et al., 2016). The role of LRRK2 protein is incompletely understood; it possesses kinase and GTPase activities, and has been associated with autophagy, mitochondrial functions, and microtubule/cytoskeletal dynamics (Wallings et al., 2015; Esteves and Cardoso, 2016; Kang and Marto, 2016). Expression of the most common PD-associated *LRRK2* mutation, G2019S (Kachergus et al., 2005), may uncouple mitochondrial oxidative phosphorylation (Mortiboys et al., 2010; Papkovskaia et al., 2012) and increase intracellular ROS production *in vitro* (Pereira et al., 2014). These findings suggest that this *LRRK2* mutation might increase CNS oxidative stress in healthy individuals, and/or contribute to oxidative stress in PD patients carrying this mutation. The interaction between LRRK2 and oxidative stress may be bidirectional, because oxidative stress has been shown to promote *in vitro* dephosphorylation of LRRK2 at Ser^910^/Ser^935^, causing loss of its ability to bind to 14-3-3 proteins (Mamais et al., 2014). 14-3-3 proteins bind to serine/threonine-phosphorylated residues, often functioning as direct regulators of the target proteins to which they bind (reviewed by Tzivion et al., 2001). Because LRRK2’s binding to 14-3-3 controls its cellular localization, decrease in this binding results in cellular translocation of LRRK2, causing it to accumulate within cytoplasmic pools (Dzamko et al., 2010; Mamais et al., 2014).

8-hydroxy-2′-deoxyguanosine (8-OHdG) (Valavanidis et al., 2009) and 8-isoprostane (8-ISO) (Morrow et al., 1992) are commonly measured oxidative stress markers. In contrast to these direct indicators of oxidative stress, total antioxidant capacity (TAC) has been suggested to be a possible indirect marker for this condition (Mandrioli et al., 2006). TAC includes antioxidant activities of non-enzymatic low molecular weight substances such as glutathione, ascorbic acid, uric acid, α-tocopherol, and coenzyme Q (Alho et al., 1998; Bartosz, 2003); a reduction in TAC could alter the balance between ROS levels and mechanisms which protect against their cytotoxic effects, in favor of oxidative damage. The concentration of 8-OHdG has been reported to be increased in PD cerebrospinal fluid (CSF) (Kikuchi et al., 2002; Abe et al., 2003; Gmitterová et al., 2009; Isobe et al., 2010). We found no reports of 8-ISO or TAC measurements in PD CSF; however, 8-ISO in the PD SNC has been reported to be unchanged (Fessel et al., 2003), while TAC in the PD SNC is reported to be decreased (Sofic et al., 2006). The primary objective of this study was therefore to compare the concentrations of 8-OHdG, 8-ISO, and TAC in CSF between *LRRK2* PD, sPD, *LRRK2* CTL, and CTL subjects. Our secondary objective was to examine the associations in these individuals of the CSF oxidative stress markers and TAC with cognitive functioning levels, and, in the two groups of PD patients, with clinical disease duration and progression.

## Materials and Methods

### Study Subjects

The subjects who participated in this study (*LRRK2* PD = 19, sPD = 31, *LRRK2* CTL = 30, CTL = 27) were recruited at St. Olav’s Hospital, Trondheim, Norway by neurologist Jan Aasly, M.D. The study was approved by the Regional Committee for Medical Research Ethics, Central Norway, for the procedures done at St. Olav’s Hospital (subject recruitment and obtaining of CSF samples), and was given exempt status by the Institutional Review Board of Beaumont Health (Royal Oak, MI, USA). All procedures relating to the subjects in the study, including obtaining of written informed consent prior to performing lumbar punctures, were performed in accordance with the Declaration of Helsinki and its subsequent amendments. Measurements of 8-OHdG, 8-ISO, and TAC were performed in the Neurology Research Laboratory at Beaumont Hospital-Royal Oak (Royal Oak, MI, USA). The diagnosis of PD was made on the basis of consensus clinical criteria (Gelb et al., 1999). The diagnosis of sPD was established by clinical history and neurological findings compatible with PD, plus lack of evidence, upon screening of whole blood-extracted DNA with standard techniques including exome sequencing (Bras and Singleton, 2011), for known PD-related mutations including those identified in the *PARK8* (*LRRK2*), *PARK2*, *PARK7*, *PINK1*, *DNAJC13*, and *SNCA* genes. (All study subjects were screened for these mutations.) All 30 *LRRK2* CTL subjects carried the G2019S gene mutation; 17 of the 19 *LRRK2* PD patients also carried this mutation, while the other two carried the N1437H mutation (Aasly et al., 2010). Unified Parkinson’s Disease Rating Scale (UPDRS) Total and Part 3 (Motor) scores were measured the same day that CSF was obtained, and Montreal Cognitive Assessment (MoCA) scores were measured the day of CSF collection or the following day. The UPDRS (Fahn et al., 1987) measures overall Parkinsonian disability; it includes 42 items, with a total possible score of 199 points (0 points = no disability; 199 points = worst possible disability). In addition to UPDRS Total scores, UPDRS Part 3, which includes 14 items and assesses motor abnormalities, is often used by clinicians to monitor PD progression. MoCA is a screening test for cognitive impairment with a 30 point scale, with scores ≥ 26 considered to be in the normal range (Nasreddine et al., 2005; Smith et al., 2007). Collection of CSF specimens followed standardized procedures used by the Parkinson’s Progression Markers Initiative (Parkinson’s Progression Markers Initiative, 2014).

### Measurements of CSF 8-OHdG, 8-ISO, and TAC

8-OHdG, 8-ISO, and TAC were measured in CSF using kits from Cayman Chemicals (Ann Arbor, MI, USA): DNA/RNA Oxidative Damage ELISA (cat. # 589320, 8-Isoprostane ELISA (cat. # 516351), and Antioxidant Assay Kit (cat. # 709001). [The DNA/RNA Oxidative Damage ELISA measures 8-OHdG, 8-hydroxyguanosine (8-OHG), and 8-hydroxyguanine; the measurements, using this kit, on CSF samples in this study will be referred to as 8-OHdG]. The detection limits for the 8-OHdG, 8-ISO, and TAC kits were stated by the manufacturer to be 30 pg/mL, 2.7 pg/mL, and 44 μM (Trolox equivalents) respectively. The standard curves for the 8-OHdG, 8-ISO, and TAC assays ranged from 10.3 pg/mL to 3,000 pg/mL, 0.8 pg/mL to 500 pg/mL, and 0.045 mM to 0.330 mM Trolox equivalents, respectively. 8-OHdG and 8-ISO were measured in duplicate after diluting CSF samples 1:4 and 1:2 respectively with EIA buffer, while TAC was measured in duplicate in undiluted CSF samples. The kits included standards used to generate standard curves, which were plotted using Softmax Pro software (version 3.0; Molecular Devices Corp., Sunnyvale, CA, USA). The concentrations of 8-OHdG, 8-ISO, and TAC in each CSF sample were then calculated with Softmax based upon where their optical density values fell on the standard curves. Concentrations of 8-OHdG, 8-ISO, and TAC used in statistical analyses were means of duplicate measurements.

### Statistical Procedures

Categorical variables were summarized by counts with percentages. Normality of the data was assessed using box plots and normal probability plots. Variables which were not reasonably normal were summarized by medians and ranges. Comparisons between the four diagnostic groups for 8-OHdG, 8-ISO, and TAC used the non-parametric Kruskal–Wallis test, while multiple comparison testing was done with a non-parametric procedure, the Dwass, Steele, Critchlow-Fligner (DSCF) procedure. *P*-values less than 0.05 were accepted as statistically significant. Spearman’s rank-order correlation coefficient (Spearman’s rho) measured the associations of 8-OHdG, 8-ISO, and TAC with subject age and MoCA scores in each of the four groups, and the associations of the two oxidative stress markers and TAC with UPDRS scores and duration of clinical disease in each of the two groups of PD patients. The SAS System for Windows version 9.3 (SAS Institute Inc., Cary, NC, USA) was used for statistical analysis, and Minitab 14 (Minitab, State College, PA, USA) was used for graphs.

## Results

### Study Subjects

The subjects in this study consisted of 66 women (62%) and 41 men (38%) with a mean age of 61.4 years (*SD* = 9.5; range 44–85). Summary statistics of demographic and clinical characteristics for the diagnostic groups are shown in **Table 1**. The four groups were well balanced for age (*p* = 0.90). Gender distribution was similar between sPD, *LRRK2* CTL, and CTL subjects (48, 40, and 37% men) but the *LRRK2* PD group had a lower proportion of men (21%). MoCA scores tended to be higher in the CTL group than in the other groups; the CTL group median was 29 while for each of the other groups the median was 27. (The *p*-value for the Kruskal–Wallis test comparing the MoCA scores between groups was <0.001; the DCSF *p*-values for all multiple comparisons involving the control group were ≤0.022.) UPDRS Total and Part 3 (Motor) scores were similar between the sPD and *LRRK2* PD patients, but the median duration of clinical disease was longer for *LRRK2* PD patients (5 years) than for sPD patients (3 years) (*p* = 0.04).

**Table 1:** Summaries of demographic and clinical characteristics for diagnostic groups.

Variable	*LRRK2* PD (*n* = 19)	sPD (*n* = 31)	*LRRK2* CTL (*n* = 30)	CTL (*n* = 27)
Age, years Mean (SD)	61.4 (10.5)	60.5 (7.9)	62.3 (10.7)	61.5 (9.3)
% Males	21	48	40	37
MoCA scores Median (range)	27 (16-29)	27 (12-30)	27 (20-30)	29 (26-30)
UPDRS Total scores Median (range)	25 (20-75)	27 (16-55)	0 (0-7)	0 (0-0)
UPDRS Part 3 scores Median (range)	19 (13-49)	20 (12-39)	0 (0-7)	0 (0-0)
Clinical duration, years Median (range)	5 (0–21)	3 (0–25)	NA	NA


*The four groups were well matched for age and gender distribution, except that the *LRRK2* PD group had a lower proportion of males. Median MoCA scores were highest in the CTL group (MoCA scores 26–30 are considered to be normal, while scores below 26 suggest cognitive impairment). UPDRS scores were similar between the PD groups (increasing UPDRS scores indicate worsening Parkinsonian disability), while the duration of clinical disease was slightly longer for *LRRK2* PD than for sPD patients. (*LRRK2* PD, Parkinson’s disease patients with *LRRK2* mutations; sPD, sporadic Parkinson’s disease patients; *LRRK2* CTL, healthy control subjects with *LRRK2* mutations; CTL, healthy control subjects without known PD-associated mutations; MoCA, Montreal Cognitive Assessment; UPDRS, Unified Parkinson’s Disease Rating Scale; NA, not applicable).*

### CSF 8-OHdG, 8-ISO, and TAC Concentrations

**FIGURE 1:**
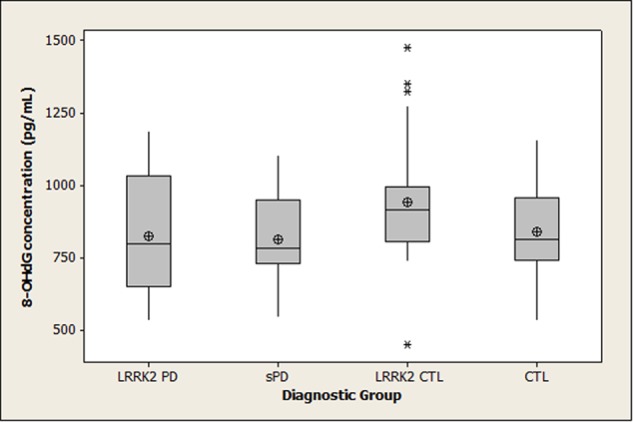
**Distribution of 8-OHdG concentrations in diagnostic groups.** Means (circle), medians (line through center of box), upper and lower quartiles (upper and lower borders of box, respectively), most extreme non-outlier values (lines extending from box), and outliers (asterisks) are shown for 8-OHdG concentrations in CSF specimens from *LRRK2* PD, sPD, *LRRK2* CTL, and CTL subjects. The Kruskal–Wallis *p*-value for the overall test of between-group differences for 8-OHdG was 0.04. The only pairwise difference which achieved *p* < 0.05 with the DSCF procedure was the comparison of 8-OHdG between the *LRRK2* CTL and sPD groups (*p* = 0.03). (*LRRK2* PD, Parkinson’s disease subjects carrying *LRRK2* gene mutations; sPD, sporadic Parkinson’s disease; *LRRK2* CTL, healthy control subjects carrying PD-associated *LRRK2* gene mutations; CTL, healthy control subjects lacking detectable PD-associated gene mutations).

**FIGURE 2:**
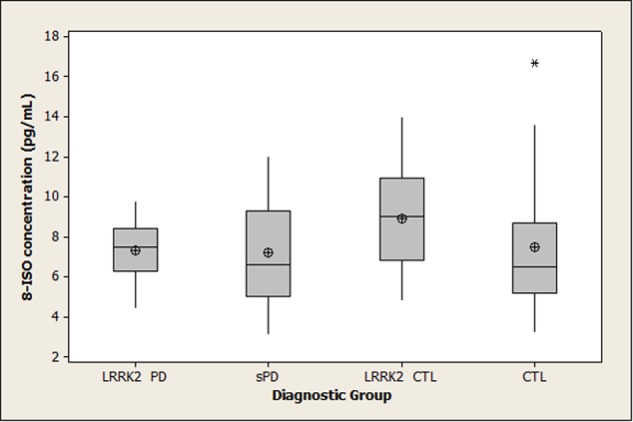
**Distribution of 8-ISO concentrations in diagnostic groups.** Means (circle), medians (line through center of box), upper and lower quartiles (upper and lower borders of box, respectively), most extreme non-outlier values (lines extending from box), and outliers (asterisks) are shown for 8-ISO concentrations in CSF specimens from *LRRK2* PD, sPD, *LRRK2* CTL, and CTL subjects. The Kruskal–Wallis *p*-value for the overall test of between-group differences for 8-ISO was 0.03. None of the pairwise differences had *p*-values less than 0.05 using the DSCF procedure (*p* = 0.06 for comparison of 8-ISO levels between *LRRK2* CTL and sPD). (*LRRK2* PD, Parkinson’s disease subjects carrying *LRRK2* gene mutations; sPD, sporadic Parkinson’s disease; *LRRK2* CTL, healthy control subjects carrying PD-associated *LRRK2* gene mutations; CTL, healthy control subjects lacking detectable PD-associated gene mutations).

**FIGURE 3:**
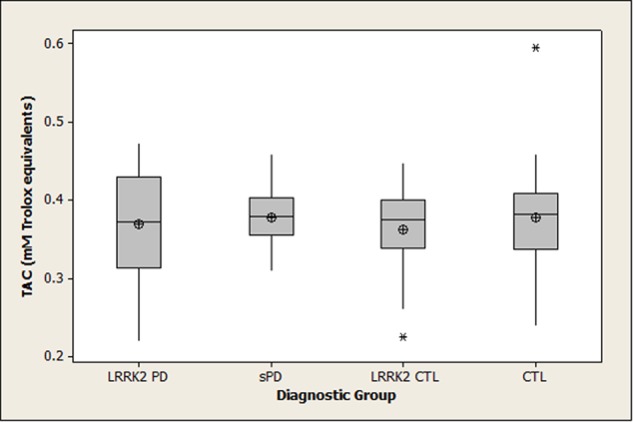
**Distribution of TAC concentrations in diagnostic groups.** Means (circle), medians (line through center of box), upper and lower quartiles (upper and lower borders of box, respectively), most extreme non-outlier values (lines extending from box), and outliers (asterisks) are shown for TAC concentrations in CSF specimens from *LRRK2* PD, sPD, *LRRK2* CTL, and CTL subjects. The Kruskal–Wallis *p*-value for the overall test of between-group differences for TAC was 0.82. (*LRRK2* PD, Parkinson’s disease subjects carrying *LRRK2* gene mutations; sPD, sporadic Parkinson’s disease; *LRRK2* CTL, healthy control subjects carrying PD-associated *LRRK2* gene mutations; CTL, healthy control subjects lacking detectable PD-associated gene mutations).

Summary statistics for 8-OHdG, 8-ISO, and TAC concentrations in the four diagnostic groups are shown graphically in boxplots in **Figures 1–3**. The Kruskal–Wallis *p*-values for overall tests of between-group differences were 0.04 for 8-OHdG, 0.03 for 8-ISO, and 0.82 for TAC. The median values for 8-OHdG and 8-ISO levels were higher in the *LRRK2* CTL group than in the other groups (by 12–16% for 8-OHdG and by 20–38% for 8-ISO), but the only pairwise difference which was significant at the 0.05 level with the DCSF procedure was the comparison of 8-OHdG concentrations between the *LRRK2* CTL and sPD groups (*p* = 0.03). [The *p*-values for comparing 8-ISO levels between the *LRRK2* CTL and each of the other three groups were < 0.20, including 0.06 for the comparison between the *LRRK2* CTL and sPD groups. Conversely, all *p*-values for pairwise comparisons between the other three groups (i.e., excluding the *LRRK2* CTL group) for 8-OHdG and for 8-ISO were > 0.70]. In contrast to 8-OHdG and 8-ISO, the median values for TAC were extremely similar between groups, with a difference of only 2.4% between the largest and smallest values.

### Correlations in All Diagnostic Groups between 8-OHdG, 8-ISO, TAC, Age, and MoCA Scores

The Spearman rank-order correlations between the pairs of variables examined in all four diagnostic groups (8-OHdG, 8-ISO, TAC, age, and MoCA scores) are shown in **Table 2** with their respective *p*-values. The concentrations of both oxidative stress markers were positively correlated with age in the control groups, and 8-OHdG was positively correlated with age in the *LRRK2* PD subjects. 8-ISO was negatively correlated with MoCA scores in *LRRK2* PD, *LRRK2* CTL, and CTL subjects, while TAC was positively correlated with MoCA scores in these groups. MoCA scores were negatively correlated with age in *LRRK2* CTL, sPD, and CTL subjects.

**Table 2:** Correlations in all diagnostic groups between 8-OHdG, 8-ISO, TAC, age, and MoCA scores.

Association	*LRRK2* PD	sPD	*LRRK2* CTL	CTL
8-OHdG vs. age	0.51 (0.02)	0.13 (0.48)	0.63 (0.0002)	0.41 (0.03)
8-ISO vs. age	-0.21 (0.39)	-0.18 (0.34)	0.36 (0.05)	0.45 (0.02)
TAC vs. age	-0.17 (0.48)	-0.06 (0.76)	0.12 (0.52)	-0.19 (0.35)
MoCA vs. age	-0.03 (0.89)	-0.47 (0.008)	-0.51 (0.004)	-0.31 (0.12)
8-OHdG vs. 8-ISO	-0.21 (0.39)	0.37 (0.04)	0.28 (0.13)	0.30 (0.13)
8-OHdG vs. TAC	-0.16 (0.52)	0.05 (0.80)	-0.12 (0.52)	-0.02 (0.91)
8-ISO vs. TAC	-0.28 (0.25)	-0.03 (0.86)	0.05 (0.77)	-0.20 (0.32)
8-OHdG vs. MoCA	-0.18 (0.46)	-0.20 (0.27)	-0.29 (0.12)	-0.11 (0.59)
8-ISO vs. MoCA	-0.35 (0.15)	-0.17 (0.37)	-0.30 (0.11)	-0.51 (0.007)
TAC vs. MoCA	0.50 (0.028)	0.14 (0.46)	0.41 (0.023)	0.56 (0.003)


*Correlations between pairs of variables examined in all four diagnostic groups are shown (Spearman rho values), with *p*-values in parentheses. Both oxidative stress markers were positively correlated with age in the control groups, and 8-OHdG was positively correlated with age in *LRRK2* PD patients. 8-ISO was negatively correlated with MoCA scores in *LRRK2* PD, *LRRK2* CTL, and CTL subjects, while TAC positively correlated with MoCA scores in these groups. MoCA scores were negatively correlated with age in *LRRK2* CTL, sPD, and CTL subjects. (8-OHdG, 8-hydroxy-2′-deoxyguanosine; 8-ISO, 8-isoprostane; TAC, total antioxidant capacity; *LRRK2* PD, Parkinson’s disease patients with *LRRK2* mutations; sPD, sporadic Parkinson’s disease patients; *LRRK2* CTL, healthy control subjects with *LRRK2* mutations; CTL, healthy control subjects without known PD-associated mutations; MoCA, Montreal Cognitive Assessment).*

### Correlations in PD Patients between 8-OHdG, 8-ISO, TAC, and MoCA Scores With UPDRS Scores and Clinical Disease Duration

**Table 3** shows the Spearman rank-order correlations and their respective *p*-values for both groups of PD patients between 8-OHdG, 8-ISO, TAC, and MoCA scores with UPDRS scores and duration of clinical disease. Correlations of 8-OHdG and 8-ISO with both UPDRS scores were weak, but TAC levels were negatively correlated with UPDRS Total scores. MoCA scores were negatively correlated with UPDRS Total and Part 3 scores in sPD patients.

**Table 3:** Correlations in PD patients between 8-OHdG, 8-ISO, TAC, and MoCA scores with UPDRS scores and clinical disease duration.

Association	*LRRK2* PD	sPD
8-OHdG vs. UPDRS Total score	-0.14 (0.56)	-0.18 (0.33)
8-OHdG vs. UPDRS Part 3 score	-0.11 (0.65)	-0.22 (0.22)
8-OHdG vs. clinical duration	-0.12 (0.62)	-0.39 (0.03)
8-ISO vs. UPDRS Total score	0.10 (0.69)	0.17 (0.35)
8-ISO vs. UPDRS Part 3 score	0.14 (0.56)	0.10 (0.61)
8-ISO vs. clinical duration	0.24 (0.32)	-0.24 (0.20)
TAC vs. UPDRS Total score	-0.33 (0.17)	-0.41 (0.02)
TAC vs. UPDRS Part 3 score	-0.30 (0.21)	-0.20 (0.29)
TAC vs. clinical duration	-0.11 (0.64)	-0.37 (0.04)
MoCA score vs. UPDRS Total score	-0.30 (0.22)	-0.49 (0.005)
MoCA score vs. UPDRS Part 3 score	-0.27 (0.27)	-0.33 (0.07)
MoCA score vs. clinical duration	-0.38 (0.11)	-0.18 (0.33)


*For the two groups of PD patients, the correlations (Spearman rho values) of 8-OHdG, 8-ISO, TAC, and MoCA scores with UPDRS scores and duration of clinical disease are shown, with *p*-values in parentheses. TAC was negatively correlated with UPDRS Total scores in both PD groups. 8-OHdG and TAC were negatively correlated with clinical duration in sPD patients, and MoCA scores were negatively associated with UPDRS scores in this group. (8-OHdG, 8-hydroxy-2′-deoxy-guanosine; 8-ISO, 8-isoprostane; TAC, total antioxidant capacity; *LRRK2* PD, Parkinson’s disease patients with *LRRK2* mutations; sPD, sporadic Parkinson’s disease patients; *LRRK2* CTL, healthy control subjects with *LRRK2* mutations; CTL, healthy control subjects without known PD-associated mutations; MoCA, Montreal Cognitive Assessment; UPDRS, Unified Parkinson’s Disease Rating Scale).*

## Discussion

Previous studies of 8-OHdG and/or 8-OHG levels in PD CSF, summarized in **Table 4**, found their concentrations to be increased relative to control CSF (Kikuchi et al., 2002; Abe et al., 2003; Gmitterová et al., 2009; Isobe et al., 2010). No information was provided in these studies regarding possible PD-related gene mutations in the study subjects. The control subjects were healthy volunteers except in the study by Gmitterová et al. (2009), in which the controls were non-cognitively impaired subjects with neurological problems other than PD. In that study PD patients with cognitive impairments (indicated by Mini Mental State Examination [MMSE] scores < 27) had lower CSF 8-OHdG levels than PD patients without cognitive impairments; only when the analysis was limited to the latter group of PD patients was the difference in the levels of 8-OHdG between the PD and control subjects statistically significant.

**Table 4:** Previous studies of 8-OHdG and/or 8-OHG in PD CSF.

	Kikuchi et al., 2002	Abe et al., 2003	Gmitterová et al., 2009	Isobe et al., 2010
Study subjects	31 PD, 16 MSA, 29 controls	24 PD, 15 controls	27 PD without dementia, 21 PD with dementia, 18 LBD, 18 AD, 13 non-demented neurological controls	20 PD, 20 controls
Variable measured	“8-OHdG/8-OHG” ( = 8-OHdG + 8-OHG)	8-OHG	8-OHdG	8-OHdG
Method	ELISA (in-house)	HPLC	ELISA (IBL Transatlantic)	HPLC
Results	PD: 2.85 ± 2.43 ng/mL; MSA: 4.24 ± 3.53 ng/mL;Controls: 1.46 ± 0.83 ng/mL	PD: 288 ± 129 pM(86.1 ± 38.6 pg/mL);Controls: 97 ± 32 pM (29.0 ± 11.5 pg/mL)	PD: 1.0 ± 0.45 ng/mL; PD with dementia: 0.90 ± 0.38 ng/mL; LBD: 0.83 ± 0.25 ng/mL;AD: 0.89 + 0.47 ng/mL;Controls: 0.71 ± 0.29 ng/mL	PD: 5.8 ± 4.5 pg/mL;Controls: 1.8 ± 0.6 pg/mL
Conclusions	PD > Controls (*p* < 0.0005)	PD > Controls (*p* < 0.001)	PD without dementia > non-demented neurological Controls (*p* = 0.03)	PD > Controls (*p* < 0.0001)


*Two studies measured 8-OHdG, one measured 8-OHG, and one measured both variables. The methods for these measurements included in-house ELISA, commercial ELISA, and HPLC. Results are shown as means and SDs. The range of measurements for 8-OHdG concentrations varies widely between the studies. (PD, Parkinson’s disease; MSA, multiple system atrophy; LBD, Lewy body dementia; AD, Alzheimer’s disease; HPLC, high performance liquid chromatography).*

Our findings in the present study do not confirm the earlier reports of increased 8-OHdG in PD CSF. Methodological differences (HPLC vs. ELISA, measurement of 8-OHdG vs. 8-OHG) and/or characteristics of the PD subjects (differences in medication, age, clinical disease duration, or severity) may have contributed to these conflicting results. Chance variation as a consequence of the small sample sizes in some of the previous studies could also have been a factor; the studies of Abe et al. (2003) and Gmitterová et al. (2009) included only 15 and 13 control subjects respectively. The median concentrations of 8-OHdG in our groups were similar to those reported by Gmitterová et al. (2009) and half to one-third of those reported by Kikuchi et al. (2002); these latter two studies also used ELISA to measure 8-OHdG, whereas Abe et al. (2003) and Isobe et al. (2010) employed HPLC. The 8-OHdG levels reported by Isobe et al. (2010) were far lower than ours, while the study by Abe et al. (2003) measured 8-OHG but not 8-OHdG so no comparison can be made to our measurements.

In contrast to the findings of Gmitterová et al. (2009) discussed above, we found no evidence that cognitive deficits were associated with lower 8-OHdG levels in our PD patients. Our analysis suggested only weak correlations between MoCA scores and 8-OHdG levels for both groups of PD patients (rho values = -0.20 for sPD patients and -0.18 for *LRRK2* PD patients). A similar trend was seen with 8-ISO; the correlations between MoCA scores and 8-ISO were -0.17 for our sPD patients and -0.35 for our *LRRK2* PD patients. When we divided our two PD groups into cognitively impaired subjects (MoCA scores < 26) and non-cognitively impaired subjects (MoCA scores ≥ 26), we similarly found no evidence that cognitive deficits lowered the CSF levels of 8-OHdG or 8-ISO in either PD group (data not shown).

An intriguing finding in this study was that both 8-OHdG and 8-ISO concentrations were increased in our *LRRK2* CTL group compared to our other diagnostic groups (*p*-values for overall tests of between-group differences = 0.04 and 0.03, respectively). Although we anticipated that *LRRK2* mutations might increase CSF oxidative stress markers in both healthy individuals and in PD patients carrying these mutations, we detected the expected increase only in our *LRRK2* CTL subjects; why a similar increase was not found in our *LRRK2* PD patients is unclear. To our knowledge, this study is the first to investigate CNS oxidative stress in individuals with *LRRK2* mutations, so our findings require confirmation with a larger cohort.

Whether the increases in 8-OHdG and 8-ISO that we detected in our *LRRK2* CTL subjects (by 12–16% and 20–38%, respectively) are of biological significance is unknown. Of relevance to our results is a report that CSF concentrations of α-synuclein soluble oligomers, suggested to be the most neurotoxic α-synuclein conformation (Winner et al., 2011), are also increased in healthy subjects with PD-associated *LRRK2* mutations compared to healthy controls lacking these mutations (Aasly et al., 2014). While oxidative stress has been suggested to be a mechanism by which soluble α-synuclein oligomers may exert their neurotoxic effects (Roberts and Brown, 2015; Deas et al., 2016), we do not know if our finding of increased CSF oxidative stress markers in our *LRRK2* CTL subjects may be related to elevated CSF levels of soluble α-synuclein oligomers in these individuals.

Our other findings in this study related to TAC. The observed correlations of TAC with 8-OHdG and 8-ISO were weak, so our results do not support the suggestion of Mandrioli et al. (2006) that TAC may be an indirect indicator of oxidative stress. We found no evidence for differences in CSF TAC activity between PD patients and control subjects with or without *LRRK2* mutations. This result agrees with an earlier report that the concentration of ascorbate, the main hydrophilic antioxidant in CSF (Lönnrot et al., 1996) and main contributor to CSF TAC activity (Alho et al., 1998; Lönnrot et al., 1996), is unchanged in PD CSF (Buhmann et al., 2004). Our finding of similar CSF TAC levels between PD patients and control subjects suggests that the decreased TAC activity reported in the PD SNC (Sofic et al., 2006) may not be reflected by TAC levels in PD CSF.

We found positive correlations between CSF TAC and MoCA scores for three of our groups (*LRRK2* PD, *LRRK2* CTL, and CTL subjects). This suggests that TAC in CSF may decrease as MoCA scores decrease (e.g., as cognitive deficits develop), regardless of the presence or absence of PD. This result is similar to the finding by Bassett et al. (1999) that CSF antioxidant capacity is reduced in patients with Alzheimer’s disease.

Our TAC results included some PD-specific findings. TAC was negatively correlated with UPDRS Total scores in both groups of PD patients. Because an increase in UPDRS scores indicates worsening Parkinsonian disability, these correlations suggest the possibility that TAC may decrease in the CNS during the progression of PD.

The numbers of CSF samples available to us were a limitation with respect to our ability to identify the locations of group differences for 8-OHdG, 8-ISO, and TAC with the DCSF procedure, and the associations between their concentrations and the other variables we examined. A second limitation is that the concentrations of these oxidative stress markers in our PD patients, although not increased compared to our control subjects, could potentially have been influenced by the prooxidative effect of their levodopa therapy (Buhmann et al., 2004).

We conclude that the concentrations of oxidative stress markers in CSF may be increased in healthy individuals with PD-associated *LRRK2* mutations. If our findings in this study can be confirmed with larger group sizes, then the possibility should be considered that an increase in CNS oxidative stress in individuals carrying these mutations may contribute to their risk for developing PD. We also conclude that TAC levels may decrease during the progression of PD, and in the presence of impaired cognitive functioning irrespective of PD status.

## Author Contributions

DL directed the study and prepared the manuscript. AK performed the assays, collated the data, and reviewed the manuscript. MC performed the statistical analysis and assisted with manuscript preparation. JA recruited the patients, collected the CSF samples, and reviewed the manuscript. PL assisted with manuscript preparation.

## Conflict of Interest Statement

The authors declare that the research was conducted in the absence of any commercial or financial relationships that could be construed as a potential conflict of interest.
